# Does doing housework keep you healthy? The contribution of domestic physical activity to meeting current recommendations for health

**DOI:** 10.1186/1471-2458-13-966

**Published:** 2013-10-18

**Authors:** Marie H Murphy, Paul Donnelly, Gavin Breslin, Simon Shibli, Alan M Nevill

**Affiliations:** 1 Sport & Exercise Sciences Research Institute, University of Ulster, Newtownabbey, Co. Antrim BT37 0QB, UK; 2 Sport Northern Ireland, Belfast, UK; 3 Sport Industry Research Centre, Sheffield Hallam University, Sheffield, UK; 4 School of Sport, Performing Arts and Leisure, University of Wolverhampton, Walsall, UK

## Abstract

**Background:**

Recent lifestyle approaches to physical activity have included the promotion of domestic physical activities such as do-it-yourself or home maintenance, gardening and housework. Although it is acknowledged that any activity is better than none, there is a danger that those undertaking domestic ‘chores’ may assume that this activity is moderate intensity and therefore counts towards this 150 minute per week target The purpose of this paper was to report the contribution domestic physical activity makes to total weekly physical activity and the relationship between domestic physical activity and leanness in the Northern Ireland population.

**Methods:**

4563 adults participated in this cross-sectional survey of physical activity behaviour. Data were collected through face-to-face interviews using computer assisted personal interviewing. Gender and age group differences in domestic MVPA activity and the ratio of domestic to total MVPA were explored using non-parametric Kruskal-Wallis tests. Self-reported volume and intensity of physical activity (in bouts of 10 minutes or more) in the home and self-reported height and weight were used to determine the association between domestic physical activity and leanness using an ANCOVA having controlled for age, gender, socio-economic and smoking status.

**Results:**

42.7% of the population report levels of physical activity which meet or exceed the current United Kingdom recommendations. Domestic physical activity accounts for 35.6% of the reported moderate to vigorous physical activity (MVPA). For women, if domestic physical activity was excluded from total MVPA, only 20.4% would be deemed to meet current recommendations. Time spent in domestic physical activity at moderate or vigorous intensity was found to be negatively associated with leanness (P = 0.024), [R Squared = .132 (Adjusted R Squared = .125)].

**Conclusions:**

Domestic physical activity accounts for a significant proportion of self-reported daily MVPA particularly among females and older adults however such activity is negatively associated with leanness suggesting that this activity may not be sufficient to provide all of the benefits normally associated with meeting the physical activity guidelines.

## Background

Current United Kingdom physical activity guidelines suggest that adults should undertake 150 minutes of moderate intensity physical activity per week [1]. Despite the health benefits associated with physical activity more than half of the population fail to meet the current recommended activity levels known to enhance health [2,3]. In an effort to encourage sedentary individuals to engage in at least some physical activity, emphasis has shifted from promoting structured forms of exercise and physical activity to lifestyle activities which can form part of a normal daily routine. This shift is based on the assumption that promoting such activity will persuade more people to become active and eventually reach levels of physical activity which meet the current guidelines. Accordingly, physical activity promotional campaigns have encouraged people to look for opportunities during normal daily life to incorporate activity into their daily routines [4] (http://www.nhs.uk/Change4Life/Pages/daily-activity-tips.aspx). This lifestyle approach to physical activity includes the promotion of domestic physical activities such as do-it-yourself or home maintenance, gardening and housework. Although cross-sectional and cohort studies have shown that domestic physical activity is associated with reduced all-cause mortality the evidence for an association between this type of activity and cardiovascular disease risk has been equivocal [5,6]. An analysis of survey data from 30 European countries has shown that domestic physical activity is only weakly associated with measures of health (self-rated health and BMI) compared to leisure time physical activity [7].

Besson and colleagues (2008) have shown that while intense domestic physical activities (>5METS) are associated with a reduction in all-cause mortality among older adults when these more intense activities are removed from the analysis the association between domestic activity and all-cause mortality disappears highlighting the importance of intensity of activity as an important mediator of the health benefits of activity. Although it is widely acknowledged that any activity is better than none, particularly when such activity replaces sedentary behaviours, there is a danger that those undertaking routine domestic ‘chores’ may assume that this activity is moderate intensity and therefore counts towards this 150 minute per week target. While some laboratory studies have shown that certain domestic physical activities such as sweeping, window cleaning, vacuuming and lawn mowing performed at a self-selected pace are moderate intensity for middle aged or older women there is large variability between individuals in the intensity at which such activities are performed [8]. If domestic physical activity is widely promoted as health enhancing and individuals believe that such activity is likely to result in improved health it may displace other activity known to be moderate intensity and more unequivocally linked to reduced disease risk. This issue may be particularly important for women – as domestic physical activity has been shown to make a larger contribution to total physical activity in females [9].

Excess body weight for height is now regarded as a significant population health issue with the proportion of individuals classified as overweight or obese rising in most developed countries [10]. Being overweight or obese is now recognised as an important risk factor for many chronic diseases [11] and has been shown to increase all-cause mortality while leanness is associated with reduced risk [12]. Cross-sectional studies have shown strong inverse associations between physical activity and body weight [13]. However, when observations are limited to domestic physical activity there is often little or no association with overweight and obesity [14].

The aims of this paper are to determine the contribution of domestic physical activity to meeting current physical activity guidelines in the Northern Ireland population, and to explore the relationship between domestic physical activity and leanness in this population.

## Methods

### Design

This study is based on secondary analysis of a cross-sectional survey of Northern Irish adults conducted in 2009/10 and known as the Northern Ireland Sport and Physical Activity Survey (SAPAS). This was the first nationwide survey specifically on sport and physical activity, commissioned by Sport Northern Ireland and carried out by an independent market research agency (IPSOS Mori). 4653 adults (aged 16+) completed face-to-face interviews conducted in their homes using computer assisted personal interviewing. This study involved the mining of an existing data set collected by Ipsos MORI who adhere to a code of conduct that encompasses ethical and legislative principles in the UK. Permission to use the data was granted by Sport Northern Ireland. The authors did not collect the data and therefore did not require ethical approval.

### Sample

The sampling procedures ensured proportionality with the Northern Ireland population based on estimates of the number of residents aged 16 or older provided by the Census Office for Northern Ireland (1.4 million). The sample was stratified by local council area (26 strata) and random samples of households, selected from the Royal Mail’s Postal Address File, were drawn within each stratum. The sampling fraction was the same for each council area except for Belfast and Derry the two major cities, where oversampling was used to facilitate detailed analysis. Within selected households, one adult was randomly selected using the last birthday rule. Participants completed a detailed interview (average time - 30 minutes) with a trained interviewer. 4,663 interviews were completed, representing a response rate of 54.6%. The dataset was weighted to control for the oversampling in the Belfast and Derry strata, differences in household size, and to ensure that the age/gender closely matched that of the adult population of Northern Ireland.

### Survey description

The survey instrument was designed in partnership with Sport Northern Ireland and was cognitively tested and piloted by the market research agency. The survey was based on the Active People Survey (Sport England) conducted annually by Sport England since 2005-06. The 30 minute interview collected data on participation in sport and physical activity, perceived health and happiness, fruit and vegetable intake, alcohol consumption and smoking habits as well as sociodemographic information. The list of physical activities was taken from the Department of Culture Media and Sport (DCMS) and Sport England (Ipsos MORI).

Piloting and cognitive testing consisted of interviews with 30 respondents covering both genders and a wide range of ages, employment statuses and levels of sport participation. A mix of ‘think aloud’ (whereby respondents are instructed to verbalise their thought processes as they answer the survey questions) and ‘verbal probing’ techniques were employed, which were adapted to suit individual respondents (with both concurrent and retrospective probing). As a result of this, some survey items were amended to maximise recognition, recall and decision-making by respondents.

### Outcome measures

#### Domestic physical activity

Participants were asked to recall how long (in minutes) they spent walking and cycling, being physically active at work and in or around the home, and participating in sport during the previous 7 days. For each activity participants were asked to classify the intensity of physical activity depending on whether or not participation raised breathing or heart rate and to report only bouts of 10 minutes or more.

Participants were asked about any physical activity in the home (domestic physical activity) that raised breathing rate over the last 7 days. Domestic physical activity was classified into 4 categories; housework, DIY, gardening or other activity. Total time spent in domestic physical activity in bouts of 10 minutes or more was reported in minutes. For each activity, participants were asked to report whether the effort they put into the activity was “usually enough to make them out of breath or sweat”. On the basis of this assessment, activity was classified as low intensity physical activity or moderate to vigorous intensity physical activity (MVPA).

### Body mass index

Self-reported height and weight were used to calculate Body Mass Index (BMI). The inverse of Body Mass Index (iBMI) was calculated by the formula height (cm) 2/weight (kg). We chose iBMI as our response variable as this ratio has been shown to be more linearly related to, and hence able to explain more of the variance in, percentage body fat. It also has the advantage of being more symmetric, better approximated by the normal distribution and hence more suitable than BMI in detecting differences in fatness/leanness when adopted in statistical/epidemiological studies [13].

### Statistical analyses

Because time spent doing moderate to vigorous domestic physical activity and the percentage of time reported doing domestic MVPA activity as a ratio of total MVPA were not normally distributed, gender and age group differences in both variables were explored using non-parametric Kruskal-Wallis tests of significance.

We also explored the effect of various sources of physical activity (at work, at home, sporting, cycling, and walking activities) on iBMI using ANCOVA, having controlled for the confounding effects of age, gender, socio-economic and smoking status. The categorical variables (gender, age group, socio-economic and smoking status) were entered as fixed factors and the continuous variables (time spent at various types of exercise) were incorporated as continuous linear covariates. Time spent at various types of exercise were entered initially as time spent at any level of exercise intensity and subsequently as time spent at a moderate or higher level of exercise intensity only.

## Results

The mean duration (mins) of weekly moderate to vigorous domestic physical activity and the relative contribution of domestic activity to total MVPA (%) by age and gender is shown in Table 1.

**Table 1:** Mean (SEM) amount of moderate to vigorous intensity physical activity and percentage contribution of domestic activity to total MVPA in 4623 adults

	**Male**			**Female**		
**Age**	**N**	**MVPA from domestic activity (mins/wk)**	**% of total MVPA from domestic activity**	**N**	**MVPA from domestic activity (mins/wk)**	**% of total MVPA from domestic activity**
16-20	134	50.4 (23.6)	8.1 (3.4)	149	59.3 (22.4)	19.1 (3.2)
21-30	264	74.1 (16.8)	13.9 (2.4)	450	170.7 (12.9)	38.2 (1.9)
31-40	343	91.1 (14.7)	16.5 (2.1)	507	175.4 (12.1)	41.1 (1.8)
41-50	345	104.2 (14.7)	20.4 (2.1)	421	154.8 (13.3)	36.3 (1.9)
51-60	317	112.3 (15.3)	25.7 (2.2)	376	164.8 (14.1)	38.7 (2.0)
61-70	287	90.0 (16.1)	28.2 (2.3)	369	116.3 (14.2)	35.5 (2.1)
71+	274	72.0 (16.5)	19.1 (2.4)	400	64.0 (13.7)	24.0 (2.0)

A total of 1989 respondents (42%) were found to meet the current guidelines of 150 minutes of moderate to vigorous physical activity per week. In this group domestic physical activity accounts for between 11 and 73% of their reported moderate to vigorous physical activity (see Table 2a). Of the 1989 respondents who meet the guidelines 1321 (66.4%) report at least 10 minutes or more of domestic physical activity per week (Table 2b). The percentage contribution of domestic physical activity for those meeting the current guidelines by age and gender are shown in Table 2a and b. Females and older individuals reported higher levels of domestic MVPA than their male and younger counterparts (*both P < 0.001*).

**Table 2:** a and b Mean (SEM) contribution of domestic physical activity to total MVPA for all respondents meeting current physical activity guidelines (N = 1989) and for those meeting guidelines and reporting at least 10 mins of MPVA from domestic physical activity (N = 1321)

	**% of reported total MVPA from domestic activity sources**			
	a. All respondents meeting guidelines MVPA >150 mins/wk (n = 1989)		b. Respondents >10 min/wk domestic MVPA and meeting guidelines MVPA >150 mins/wk (n = 1321)	
Age	Male n = 1074	Female n = 909	Male n = 472	Female n = 847
16-20	11.2 (4.0)	24.3 (4.5)	44.6 (6.8)	49.3 (5.4)
21-30	13.8 (2.9)	52.3 (2.4)	39.2 (4.1)	67.7 (2.3)
31-40	20.6 (2.6)	52.6 (2.2)	37.0 (3.0)	65.5 (2.1)
41-50	28.0 (2.7)	53.7 (2.7)	49.2 (3.0)	70.2 (2.6)
51-60	39.7 (3.2)	62.0 (2.9)	62.2 (3.4)	72.7 (2.7)
61-70	47.4 (3.7)	71.3 (3.5)	72.9 (3.9)	83.4 (3.2)
71+	49.0 (5.3)	73.8 (4.8)	76.7 (5.7)	82.1 (4.3)

When domestic physical activity is included 42% of the population (men 46% women 40%) meet current physical activity recommendations of ≥ 150 mins of MVPA per week. For women, if domestic physical activity was excluded from their total MVPA, only 20.4% would be deemed to meet current recommendations

When we explored the effect of various sources of physical activity (at work, at home/domestic, sporting, cycling, and walking activities) on iBMI, having controlled for the confounding effects of age, gender, socio-economic and smoking status, time spent in domestic physical activity at moderate to vigorous intensity was found to be a negative covariate (P = 0.024), [R Squared = .132 (Adjusted R Squared = .125)].

## Discussion

In this cross-sectional study over two thirds of participants reported taking part in at least one 10 minute bout of domestic physical activity which they rated as moderate to vigorous in intensity (usually enough to make them out of breath or sweat). This compares with the Scottish Health Survey where, 42.9% of men and 39.8% of women reported participating in 20 minutes of intense domestic physical activity [6]. The differences between the two findings are likely to be due to differences in the threshold used for measuring physical activity. The 20 minute threshold employed in the Scottish survey is likely to have resulted in a slightly lower proportion of respondents who were categorised as having reported moderate to vigorous intensity domestic physical activity. Irrespective of these differences our finding underscores the prevalence of domestic physical activity in the adult population and the degree to which such activity is perceived to be at least moderate intensity. The prevalence of moderate to vigorous domestic physical activity was not equally distributed throughout the population. Domestic physical activity accounted for 34.9 (+0.8)% of all reported MVPA for women whereas among men it only accounted for 19.8 (+0.8)% of total MVPA. This gender difference is in keeping with other self-report surveys of physical activity and probably reflects both the greater role that women traditionally play in completing household chores and/or the degree to which this activity is perceived as moderate intensity. In both genders the contribution of domestic physical activity to self-reported MVPA increases with age. For females over 21 years who reported at least 10 mins of moderate to vigorous domestic physical activity, this source accounts for more than 50% of total MVPA. Indeed if domestic physical activity was eliminated from the analysis less than 21% of the females taking part in this survey would be deemed to have met current physical activity recommendations. This finding reflects the results of analysis from Scottish and Australian surveys where eliminating domestic physical activity significantly reduced the proportion of the population who were deemed to meet current physical activity guidelines [9,15].

In our analysis, domestic MVPA was negatively associated with leanness. One explanation for the negative association observed in this analysis is that less lean individuals may self-report domestic activities as being more intense than their leaner counterparts. Domestic activity often involves smaller muscle groups and isometric contraction which are more likely to cause fatigue and result in perceived exertion without resulting in the volume of energy expenditure likely to alter body composition. Moreover it could be speculated that fatter respondents report greater levels of moderate to vigorous intensity domestic physical activity because of their larger body mass and the difficulties associated with performing domestic tasks. The finding that domestic physical activity is inversely related to leanness is counter-intuitive. Irrespective of the intensity of domestic physical activity additional physical activity incurs additional energy expenditure and should, from a physiological perspective, be associated with greater leanness assuming energy intake is constant [16]. We speculate that those reporting the highest levels of MVPA through domestic physical activity are either over-estimating the intensity or duration of this physical activity or are over-compensating for the energy expended in such physical activity through energy intake. As suggested above, if individuals perceive that they are undertaking significant amounts of moderate or vigorous intensity physical activity through domestic activities they may be inclined to increase their energy intake which may, over time, contribute to an increase in fatness.

Recent findings from the Scottish Health Survey suggest that domestic activities do not protect against cardiovascular disease [6]. In a prospective cohort study of English older adults (>63ys) [5] reported a 19% reduction of risk of all-cause mortality attributable to domestic physical activity and an inverse association between such activity and cardiovascular mortality independent of other sources of physical activity. However when the most intense domestic physical activities (>5METS) such as mowing the lawn, digging and stair climbing were excluded the association with all-cause mortality disappeared underlining the importance of intensity of domestic physical activity for health benefit.

Given the emergence of sedentary behaviour as a risk factor for many chronic diseases the promotion of any physical activity, irrespective of intensity, is well-founded. However if public health campaigns are designed to encourage people to meet current physical activity guidelines then caution is needed in the promotion of domestic physical activity as this may be insufficient to evoke some of the favourable alterations that lead to improved health and decreased risk. Where domestic physical activity is promoted for such purposes clear messages on the intensity required should be provided so that individuals can distinguish between activities which can be counted towards the current 150 mins per week target and those which may be useful for reducing sedentary time and boosting their overall volume of physical activity but do not qualify within the current guidelines.

There are several limitations to this study including the use of self-reported physical activity, self-estimated exercise intensity and self-reported height and weight [3]. Neither energy expenditure nor dietary intake was objectively measured and therefore our explanation of the negative association between domestic physical activity and leanness remains speculative.

## Conclusions

The negative association between domestic physical activity and leanness found in this cross-sectional study suggests that those promoting physical activity for health benefit should ensure that the importance of physical activity across a wide variety of domains is emphasised and that domestic physical activity is not seen as the main method by which sedentary individuals are encouraged to meet current physical activity guidelines.

## Competing interests

The authors declare that they have no competing interests.

## Authors’ contributions

SS and PD were responsible for survey design and collection. MM, GB and AN were responsible for drafting the manuscript. MM and AN were responsible for data analysis. All authors read and approved the final manuscript.

## Pre-publication history

The pre-publication history for this paper can be accessed here:

http://www.biomedcentral.com/1471-2458/13/966/prepub
